# Human monocytes undergo functional re-programming during differentiation to dendritic cell mediated by human extravillous trophoblasts

**DOI:** 10.1038/srep20409

**Published:** 2016-02-09

**Authors:** Lei Zhao, Qianqian Shao, Yun Zhang, Lin Zhang, Ying He, Lijie Wang, Beihua Kong, Xun Qu

**Affiliations:** 1 Institute of Basic Medical Sciences, Qilu Hospital, Shandong University, Jinan, 250012, Shandong, P.R. China; 2 Department of Obstetrics and Gynecology, Qilu Hospital, Shandong University, Jinan, 250012, Shandong, P.R. China

## Abstract

Maternal immune adaptation is required for a successful pregnancy to avoid rejection of the fetal–placental unit. Dendritic cells within the decidual microenvironment lock in a tolerogenic profile. However, how these tolerogenic DCs are induced and the underlying mechanisms are largely unknown. In this study, we show that human extravillous trophoblasts redirect the monocyte-to-DC transition and induce regulatory dendritic cells. DCs differentiated from blood monocytes in the presence of human extravillous trophoblast cell line HTR-8/SVneo displayed a DC-SIGN^+^CD14^+^CD1a^−^ phenotype, similar with decidual DCs. HTR8-conditioned DCs were unable to develop a fully mature phenotype in response to LPS, and altered the cytokine secretory profile significantly. Functionally, conditioned DCs poorly induced the proliferation and activation of allogeneic T cells, whereas promoted CD4^+^CD25^+^Foxp3^+^ Treg cells generation. Furthermore, the supernatant from DC and HTR-8/SVneo coculture system contained significant high amount of M-CSF and MCP-1. Using neutralizing antibodies, we discussed the role of M-CSF and MCP-1 during monocyte-to-DCs differentiation mediated by extravillous trophoblasts. Our data indicate that human extravillous trophoblasts play an important role in modulating the monocyte-to-DC differentiation through M-CSF and MCP-1, which facilitate the establishment of a tolerogenic microenvironment at the maternal–fetal interface.

During pregnancy, maternal immune adaptation is naturally induced to avoid rejection of the fetal–placental unit, which expresses paternal histocompatibility antigens. Under the conditions of the breach of this immune adaptation, the placenta and fetus will be attacked as a foreign organ transplant resulting in pregnancy failure. To date, although many important discoveries in development of immune tolerance have been revealed, the immunological paradox of pregnancy is still fascinating.

Dendritic cells (DCs) are the professional antigen-presenting cells (APC) that play a key role in inducing immunity as well as maintaining tolerance. Within peripheral tissues, dendritic cells can confer immune tolerance through a variety of mechanisms, such as expanding regulatory T cells, limiting the proliferation of effector T cells and inducing the apoptosis of antigen-specific T cells^1^. Several studies have demonstrated that DCs play an important role in establishing tolerant microenvironment at the maternal-fetal interface^2^,^3^, and the underlying mechanisms involve the induction of Treg cells and the expansion of CD4^+^HLA-G^+^T cell^4^. Human decidual DCs express unique phenotype^5^, and the dysregulation of DC differentiation may lead to the destruction of maternal immune tolerance, which in turn causes a negative pregnancy outcome. However, how these DCs are induced and the underlying mechanisms remain largely unknown.

Circulating monocytes have been considered as natural precursors of dendritic cell and macrophage^6^,^7^,^8^. Given their inherent plasticity, monocytes can give rise to tissue-resident macrophages and dendritic cells after tissue recruitment. In the context of pregnancy, monocytes migrate from the bloodstream into the decidua, and the differentiation and function of these cells may be shaped upon exposure to decidual microenvironment. At the maternal-fetal interface, EVTs deeply penetrate into decidual tissue and formed close contact with decidual lymphocytes at embryo implantation site^9^,^10^. The anatomical location of EVTs allows them to become a potential candidate for educating maternal dendritic cell to generate a tolerant decidual microenvironment. At present, the interaction between trophoblasts and decidual DC has been reported, showing the regulatory effect on decidual DC function through cytokine secretion and membrane molecules expression^11^,^12^. Other studies focus on the maturation process of committed DCs. One report showed that DCs co-cultured with cytotrophoblasts displayed similar levels of maturity compared with those cultured alone, and its ability to induce T cell proliferation had no significant change^13^. In contrast, a recent study showed that the interaction with trophoblast cell line Swin-71 inhibited LPS-induced upregulation of CD83 on immature DCs and suppressed the following allogeneic response stimulated by DCs^14^. However, as the main local factor from fetal part of maternal-fetal interface, the regulatory effect of EVTs on monocyte differentiation, namely monocyte-to-DC transition, remains poorly understood.

Based upon the above observations, we assume that EVTs may affect the differentiation of monocyte, leading to the induction of decidual tolerogenic DCs. On the basis of this hypothesis, in present study, using the immortalized human first-trimester EVT cell line HTR-8/SVneo^15^, which is widely used as a substitute for human primary trophoblast cells, we explored the effect of EVTs on DC differentiation by assess the phenotype and biological function of dendritic cells modulated by EVTs. Furthermore, using EVTs and DCs co-culture system and neutralizing antibody, we aimed to determine which factors were involved in the cross-talk between these cells.

## Result

### Phenotypic changes of DCs in the presence of human trophoblast cells during monocyte-to-DC differentiation

**Figure 1:**
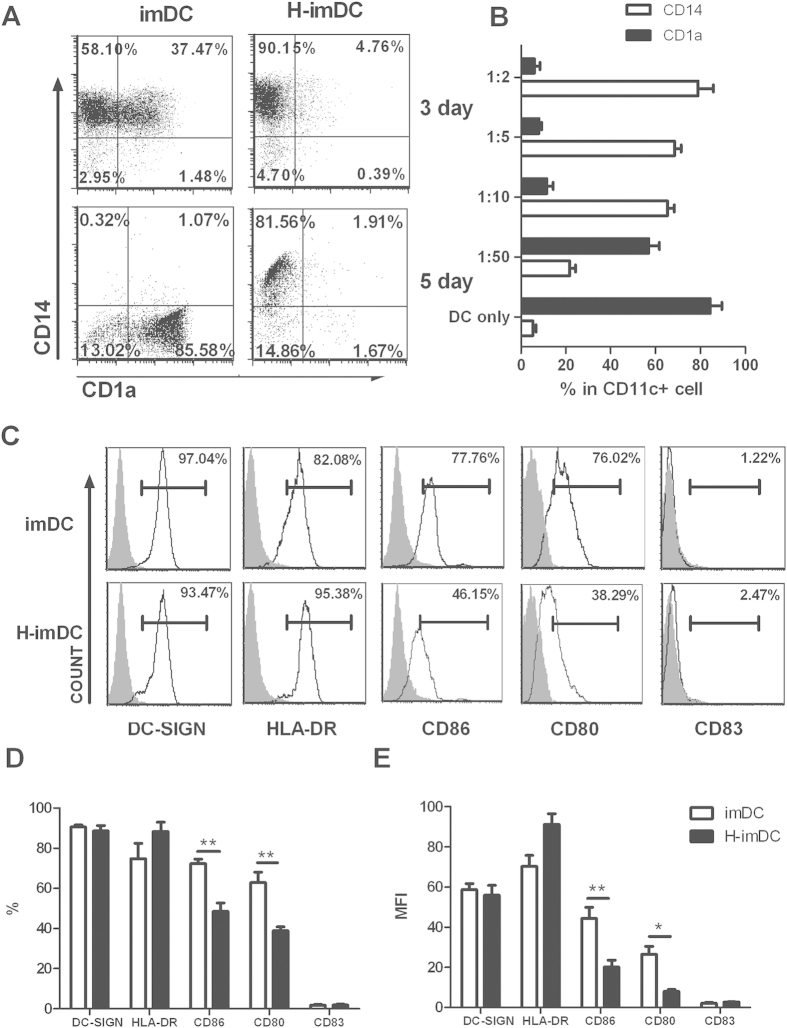
EVTs redirect the differentiation of DC to a CD1a^−^CD14^+^ phenotype. (**A**) CD14^+^ monocytes were cultured for 5 d with GM-CSF and IL-4 in the absence (imDC) or presence of seminal plasma (H-imDC). The HTR8:CD14^+^ cells ratio of 1:10 was used in coculture system. Cells obtained from day3 and day5 of culture were analyzed by flow cytometry. The expression of CD1a and CD14 was displayed gated on CD11c^+^ cells. Dot plots from a representative experiment (n = 8) are shown. (**B**)The percentages of CD1a^+^ and CD14^+^ cells in DCs in HTR-8/ monocytes cocultures at ratios ranging from 1:2 to 1:50 were assessed by flow cytometry at day 5. Experiments were performed using monocyte from 8 distinct donors. (**C**) The phenotype of imDC and H-imDC (the ratio of HTR-8:monocyte is 1:10, day5) were analyzed by flow cytometry. Representative histograms from at least 5 individual experiments are shown. (isotype control Ab: gray filled histogram; indicated mAbs: open histogram) (**D,E)** The summarized results of surface markers expression are shown as the percentage (**D**) and the mean fluorescence intensity (**E**). The bar graphs represent mean ± EM of five individual experiments (*P < 0.05; **P < 0.01)

*In vitro* cultures of human CD14^+^ monocytes with GM-CSF and IL-4 induces the differentiation of immature DCs, with characteristic marker expression, including CD1a, DC-SIGN and CD11c, whereas the expression of CD14, a monocyte marker, is lost. To examine whether human extravillous trophoblast cells influence the differentiation of DCs, CD14^+^ monocytes from PBMC of non-pregnant women were cultivated with human extravillous trophoblast cell line HTR-8/SVneo cells in the presence of GM-CSF/IL-4 to mimic decidual microenvironment. After the coculture, imDCs were harvested by mild repeated pipetting, and the expression of surface markers was examined by FACS. As shown in Fig. 1A, the significant modification was noted for the expression pattern of CD1a and CD14 in cells cultured in the presence of HTR8 cells (HTR8:CD14^+^ cells = 1:10). HTR8-modified imDCs (H-imDC) showed almost negative of CD1a expression but high CD14 expression both on day 3 and day 5 compared with control imDC. On day 5 of differentiation, H-imDCs still displayed high CD14 expression. The modulatory effect of HTR-8/SVneo cells on differentiation of DCs from CD14^+^ progenitor cells was dependent on HTR8: CD14^+^ cells ratio, with an maximum effect at a HTR8:CD14^+^ cells ratio of 1:5 and 1:10 (Fig. 1B). Considering the cells viability during 5 days coculture, we chose 1:10 in the subsequent experiments.

Monocytes do not express the pattern recognition receptor DC-SIGN^16^, and coculture with HTR8 induced comparable levels of DC-SIGN on H-imDCs with control DCs (Fig. 1C). H-imDCs showed decreased incidence of costimulatory molecules CD80 and CD86 (Fig. 1C,D), as well as the level of expression of these molecules determined by mean fluorescence intensity (MFI)(Fig. 1E). Although H-imDCs tended to increase HLA-DR expression, no sign of other mature marker was observed during differentiation process based on nearly undetectable CD83 expression on H-imDCs. The data indicate that EVTs inhibit the yield of CD1a^+^DC from monocyte, and redirect the differentiation of monocyte into a DC-SIGN^+^CD14^+^CD1a^−^ cell, which harbor similar phenotype with decidual APC^5^.

### Human extravillous trophoblasts compromised the subsequent maturation of imDCs and altered its cytokine profile

**Figure 2:**
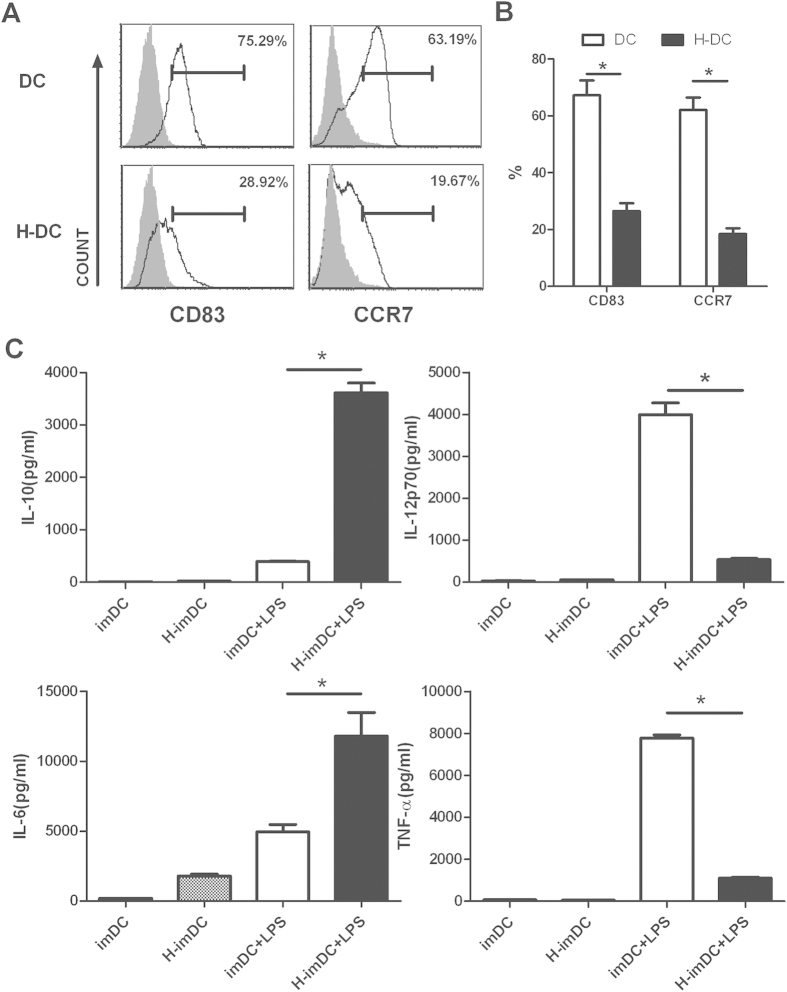
DCs differentiated in the presence of EVTs show reduced CD83 and CCR7 expression and altered cytokine profile upon stimulation by inflammatory stimuli. At day 5, cells were collected, washed and induced to mature by exposure to 100 ng/ml LPS for 48 h in the absence of HTR8, and analyzed at day 7 for CD83 and CCR7 expression. (**A**) Histograms from a representative experiment are showed (n = 5). isotype control Ab: gray filled histogram; indicated mAbs: open histogram (**B**) Graph bars indicating the percentages of CD83^+^ and CCR7^+^ cells (mean ± SEM, n = 5) are shown. (*P < 0.05) (**C**) Supernatants were collected from the indicated culture and cytokine production was evaluated by ELISA. Results represent mean ± SEM of 5 experiments (*P < 0.05).

LPS is a common maturation agent in DC studies^17^,^18^. Before LPS exposure, DCs were separated with HTR8, washed in PBS and recultured at equal densities for LPS treatment. As shown in Fig. 2A,B, expectedly, after 48h stimulation, LPS induced a strong up-regulation of maturation protein CD83 surface expression on DC. In contrast, we found that HTR8-conditioned DCs only showed a low or moderate increase of CD83 expression. Meanwhile, H-DCs harbored impaired expression of CCR7 molecule compared with control DCs, implicating the low homing capacity to draining lymph nodes. We conclude that human extravillous trophoblasts compromise the full maturation of DCs by LPS.

Because the cytokine profile of DCs is especially important in determining subsequent T cell responses, we next evaluated the cytokine profile of both DC types before and after LPS stimulation (Fig. 2C). As expected, IL-10 and IL-12 were not produced by DCs in the absence of stimulation, and both cytokines were induced by the addition of LPS. Compared with control DCs, H-DCs produced remarkable lower amounts of IL-12 and TNF-α after LPS treatment, whereas they produced higher amounts of IL-10, suggesting that H-DCs may posses immunoregulatory property. Altogether these data suggest that TLR-dependent DC maturation and cytokine secretion are markedly modified by HTR8 cells.

### DCs modulated by human extravillous trophoblasts displayed hypo-stimulatory capacity to T cells proliferation and activation

**Figure 3:**
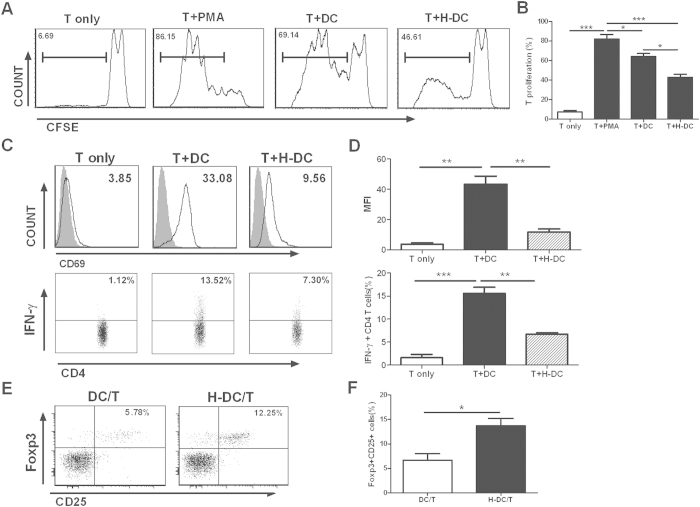
EVT-conditioned DCs show reduced capacity to prime naive T cell proliferation and activation, but enhanced capacity to induce Tregs generation. (**A**) DCs and H-DCs were harvested and washed after treated with LPS (100 ng/ml, 48 h), then seeded with CFSE-labeled allogeneic naive CD4^+^T lymphocytes at a DC/lymphocyte ratio of 1:5 in the present of IL-2(50 U/ml). The dilution of CFSE staining was analyzed at day 5 by flow cytometry. T cells cultured alone with or without PMA (10 mg/ml) were used as positive and negative controls, respectively. Histograms from a representative experiment (n = 5) are shown. (**B**) Graphical representation of data in (**A**) is shown as mean ± SEM from 3 to 5 independent experiments with consistent results. (**C**) Mature DCs and H-DCs were incubated with allogeneic naive CD4^+^T cells using a DC/lymphocyte ratio of 1:2. At day 5, cells were harvested for surface staining of CD4 and CD69. For intracellular staining of IFN-γ, cells were resuspended in fresh medium, stimulated with PMA/ionomycin for 6 h and treated with brefeldin A for the last 4 h of cultures. The representative flow cytometry pictures are depicted from a representative experiment (n = 5). Gray filled histograms, isotype control Ab staining; Solid lines, anti-CD69 Ab staining. (**D**) Summary of the proportion of CD69 (upper) and IFN-γ (lower) on CD4^+^T cells. The results are shown as mean ± SEM from 5 independent experiments. (**E**) Mature DCs and H-DCs were recovered and then cultured for 5 days with isolated naive CD4^+^T cells in the present of IL-2(50U/ml). Cells were stained with anti-CD4, anti-CD25 and anti-Foxp3. Flow cytometry dot plots showing that CD25^+^Foxp3^+^ Tregs increased in the CD4^+^T cell population were shown. (**F**) The percentage of CD25^+^Foxp3^+^ cells in the CD4^+^T cell population was summarized in the bar graph(mean ± SEM, n = 5). *p < 0.05, **p < 0.01, ***p < 0.001.

With respect to the different phenotype and cytokine profile of HTR8-conditioned DCs, we posed the question as to whether the two types of DCs possess distinct function in the controlling of T cell responses. Therefore, we investigated the functional consequences of H-DC on the allogenic T cell proliferation and activation in mixed lymphocytes cultures. DC cell numbers in HTR8-conditioned and control cultures were equalized before further functional assays. Purified CD4^+^T cells were stained with the cell surface dye CFSE and then stimulated by allogeneic control DC or H-DC. The proliferation of CD4^+^T cell in response to HTR8-conditioned DCs decreased significantly compared with those induced by control DCs(Fig. 3A,B), indicating the impaired capacity of HTR8-conditioned DCs to induce naive CD4^+^T cell proliferation.

Furthermore, HTR8-conditioned DCs led to reduced expression of activated marker CD69 and secretion of significantly lower amounts of Th1 cytokines (IFN-γ) by T cells(Fig. 3C,D). Very less Th2 cytokine (IL-4) were detected in both groups(less than 1% of the cells, data not shown), indicating the inefficiency of the monocyte derived DC and naive CD4^+^T cell coculture system to induce Th2 differentiation.

### Human extravillous trophoblasts modified DCs to promote Treg generation

Previous studies described that semi-mature tolerogenic DCs can induce Foxp3^+^CD25^+^CD4^+^ Treg expansion^19^, and human CD4^+^CD25^+^ regulatory T cells play an important role in mediation of maternal-fetal tolerance^20^. Therefore, we explored whether the priming of CD4^+^T cells by HTR8-conditioned DCs actually induced Treg cells, based on the expression of CD4, CD25 and Foxp3. As depicted in Fig. 3E,F, the frequency of Foxp3^+^CD25^+^CD4^+^ cells was significantly increased in the presence of HTR8-conditioned DCs. As control, DCs without interaction with trophoblast cells induced lower Foxp3 expression in CD4^+^CD25^+^T cells.

### Soluble factors are involved in human trophoblast cells-induced alteration of M-DC differentiation

**Figure 4:**
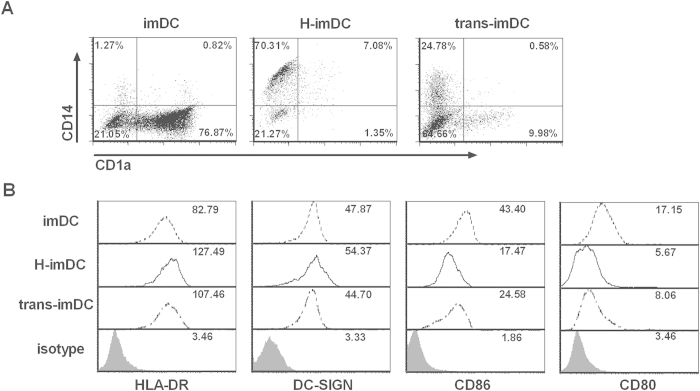
EVTs modulate the phenotype of DCs though soluble factors. CD14^+^ monocytes were cultured with GM-CSF and IL-4 in the absence (DC)or presence of HTR8(H-DC), or separated with HTR8 in transwell chamber(trans-DC). (**A**) Cells were harvested at day 5 and examined for the expression of CD1a and CD14. Representative dot plots are shown. (**B**) The phenotypes of the indicated DCs were analyzed by flow cytometry. Representative histographs from 5 individual experiments were depicted.

To investigate the effect of DCs differentiation mediated by HTR8 is dependent on direct contact or soluble factors, the transwell camber system was used. Monocytes in the upper compartment were seperated with HTR8 cells in the lower compartment. As shown in Fig. 4A, flow cytometry analysis of the CD1a, CD14 expression showed that HTR8 cells in direct contact with monocytes suppressed the CD1a upregulation and CD14 downregulation on DCs (H-imDC). Notably, when HTR8 cells were physically separated from monocytes (trans-imDC), this suppression was still observed. In addition, DCs in transwell setting showed almost comparable levels of HLA-DR, DC-SIGN, CD86 and CD80 expression compared with those in direct contact setting(Fig. 4B). In contrast, the medium with 50% volume supernatants from HTR8 cells culture had no effect on the differentiation of monocytes to DCs (data not shown).

Altogether, although direct contact between monocytes and EVTs was most powerful in impairing DC differentiation, the transwell system displayed most of the modulatory effect, suggesting that soluble factors mainly mediate the modulatory effect of DC differentiation by EVTs.

### Human EVTs and DC coculture induced high MCP-1 and M-CSF production

**Figure 5:**
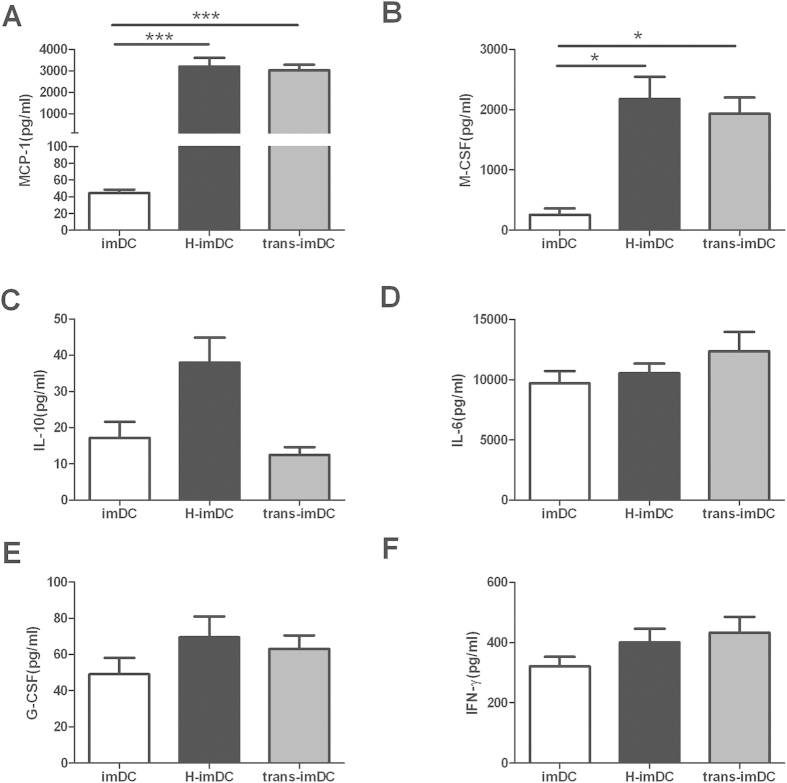
High concentration of MCP-1 and M-CSF in EVT-DC coculture system. CD14^+^ monocytes were cultured with GM-CSF and IL-4 in the absence (DC)or presence of HTR8(H-DC), or separated with HTR8 in a transwell system(trans-DC). The cell-free supernatants of different cultures were collected at day 5 of differentiation process. The concentration of MCP-1, IL-6, IL-10, G-CSF and IFN-γ were analyzed by the Bio-Plex Protein Array system. The production of M-CSF was examined by ELISA. Data are the mea ± SEM from 3 independent experiments. *p < 0.05, ***p < 0.001.

Earlier studies have demonstrated that several cytokines are known to be involved in regulatory effect on the differentiation of DCs, including IL-10, IL-6, M-CSF, IFN-γ and G-CSF^21^,^22^,^23^. Therefore, to investigate which soluble factors in the EVT/DC coculture system may be responsible for the observed modulatory effect of EVTs on DCs differentiation, cell-free supernatants from the above described cocultures and transwell system were measured by the Bio-Plex Protein Array system, and compared with those from control DCs culture. As shown in Fig. 5A–F, within the cytokines being tested, the supernatant from HTR8/DC direct coculture and transwell system contained significant higher amount of M-CSF protein. The other cytokines which have been shown to be important in DC differentiation, such as IL-10,IL-6, IFN-γ and G-CSF, showed no difference between groups. Interestingly, up to 100 fold higher amount of MCP-1 production was observed in HTR8/DC direct and indirect cocultures, which was also found in the 40 hours coculture of HTR8 and monocytes from our previous work^24^.

### Neutralization of M-CSF or MCP-1 partially abolished the modulating effect of EVTs on the DC differentiation

**Figure 6:**
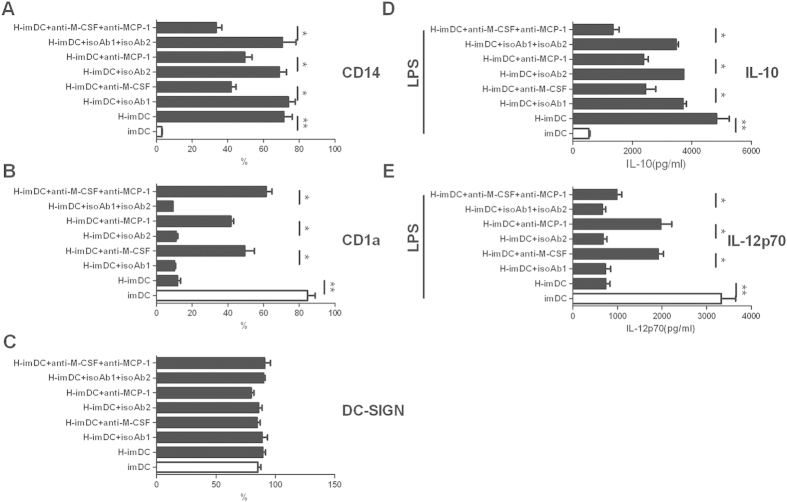
The modulating effect of EVTs on DCs is partially reversed by anti–MCP-1 and anti-M-CSF neutralizing Ab. Neutralizing Abs against MCP-1 or M-CSF or both were added in HTR8-DC coculture system during the differentation of DCs. The isotype Abs were added as control at the same time. Cells from different culture were harvested at day 5, and the expression of CD14 (**A**), CD1a (**B**) and DC-SIGN (**C**) by the indicated DCs were summarized. The production of IL-10 (**D**) and IL-12 (**E**) by the indicated DCs upon LPS stimulation were measured and shown in the bar graph. Graphs show mean ± SEM of 5 individual experiments. *p < 0.05, **p < 0.01.

To confirm the contributions of M-CSF and MCP-1 implied by the results of cytokine detection, neutralizing antibodies against these factors were used to analyze their capacity in restoring DC differentiation. As shown in Fig. 6A, in the presence of the anti-MCP-1 or anti-M-CSF neutralizing Ab, the surface-level expression of CD14 on H-DCs was significantly decreased compared with that on cells were treated with the isotype control or PBS. The anti-MCP-1 or anti-M-CSF Abs also significantly reversed the suppressive effect of the HTR8 cells on the surface expression of CD1a on DCs (Fig. 6B). DC-SIGN expression was not effect by any of Abs treatment (Fig. 6C). The corresponding isotype control Abs had no effect on any of surface marker expression. Furthermore, adding both anti-MCP-1 and anti-M-CSF Abs to the culture was modestly more effective than adding anti-MCP-1 or anti-M-CSF Abs alone on the expression of phenotypic molecules (Fig. 6A–C), suggesting that the influence of these cytokines was additive or synergistic to some extent.

In addition, the results from ELISA experiments further confirmed the role of these cytokines by showing that HTR8-conditioned DCs in the present of anti-MCP-1 or anti-M-CSF Abs or both secreted higher levels of IL-12 and lower levels of IL-10 than that in the absent of those antibodies after LPS stimulation(Fig. 6D,E). Together, based on the incomplete restoration of DCs phenotype and cytokine production, our data suggest that M-CSF and MCP-1 are involved in the modification of DC differentiation by EVTs and other factors may also play a role in this process.

## Discussion

During pregnancy, a complex and reciprocal interaction between fetal trophoblast cells and maternal immune system is required to maintain fetal-maternal tolerance. In this study, we demonstrate that human extravillous trophoblasts modulate the differentiation of monocyte-derived DCs through M-CSF and MCP-1, which promotes the induction of a tolerogenic microenvironment at the maternal–fetal interface. By using a *in vitro* model, where CD14^+^ monocyte were cocultured with EVTs to mimic decidual environment, we showed that EVTs redirected the differentiation of immature DCs toward a regulatory phenotype. DCs differentiated with HTR-8/SVneo cells were shown to be CD14^+^CD1a^−/low^ DC-SIGN^+^, which were consistent with phenotypic characteristics of decidual APC described before 5. HTR8-conditioned DCs expressed lower levels of CD86 and CD80 and their maturation was compromised upon stimulation by inflammatory stimuli. DC function was also affected by exposure of EVTs, such that we observed enhanced IL-10 production, inhibition of T cells proliferation and activation and induction of CD4^+^CD25^+^Foxp3^+^ Treg, which is in line with a role for decidual DCs in development and maintenance of fetal tolerance. we also showed, by blocking experiment, that M-CSF and MCP-1 were involved in mediating the reprogramming of DCs differentiation by EVT.

During pregnancy, the endometrium undergoes decidualization. Fetal extravillous trophoblasts come into decidua and contribute to placental anchoring, decidual cells differentiation and uterine spiral arteries remodeling. Emerging evidence has demonstrated that EVTs interact with maternal leukocytes to program their regulatory properties for maintenance of a local immunosuppressive state. It has been shown that the factors derived from placenta explants and EVTs induced homeostatic M2 macrophages^25^. In addition, the interaction of CD4^+^T cells with EVTs increased the proportion and expression level of Foxp3 within Treg^26^. Besides the modulating effect of EVTs on other decidual cell types, our results suggest that the induction of tolerogenic DCs could also be related to the unique contribution of EVT in orchestrating decidual immune response.

It has been observed that there is a unique DC-SIGN^+^APC only being found in the decidua of early human pregnancy. These cells expressed CD11c and CD14, little or no expression of CD1a^5^, as well as low levels of CD86 and CD205^27^. In our study, HTR8-conditioned DCs shared the CD1a^−^CD14^+^DC-SIGN^+^ phenotype with decidual DC-SIGN^+^APCs, which might be related to redirect the local immune response to tolerance since their function could be altered by other lymphocytes^28^. Some authors consider decidual DC-SIGN^+^ cells as macrophages based on their morphology and co-expression of CD14^29^. However, we did not confirm whether HTR8-conditioned DCs were actually macrophage or not. Based on hardly detectable CD68 and CD163 surface expression, we prefer to consider these cells as unique dendritic cells with tolerogenic features after undergoing specific differentiation process. Notably, DC and macrophages share surface markers and function in tissue, and are able to interconvert^30^. In the context of pregnancy, when treated with a cytokine cocktail, decidual CD14^+^ cells have been shown to convert to high CD83 expressing cells which can stimulate T cell proliferation with potency similar to that of monocyte–derived DCs^31^. The data raise a possibility that decidual CD14^+^ cells may convert to DC-like cells under certain circumstances, and these cells may serve as a potential pool of DC progenitors. Other group identified a new subset of tolerogenic DC at maternal-fetal interface, characterized by expression of HLA-G and ILT4 and secretion of IL-10, thus named DC-10^4^. These DC-10 might be involved in enhancing immune tolerance via promoting T regulatory type 1 (Tr1) generation^32^. In our present study, HTR8 -conditioned DCs showed potent IL-10 secretion and enhanced inducion of CD4^+^CD25^+^Foxp3^+^ Treg populations, suggesting that the interaction between EVTs and decidual myeloid cells may contribute to *in situ* generation of a set of tolerogenic DCs, including DC-SIGN^+^dDCs and DC-10.

DCs are crucial not only for initiation of adaptive immune responses but also for induction and modulation of immune tolerance. The tolerogenic capacity of DCs is determined in a large extent by their maturation status. We found that DCs differentiated with HTR8 have impaired maturation, even after being separated with HTR8 cells and stimulated by LPS. Moreover, a recent data showed that human trophoblast cells also affected the maturation process of committed immature DC^14^. In line with this, in decidua of human first-trimester pregnancy, CD83^+^DCs stay in low number and possess poor T cell stimulatory capacity in *ex vivo* mixed leukocyte reactions^33^. We speculated that EVTs may contribute to control the low CD83 expression of decidual DCs, thus sustaining a homeostatic microenvironment which facilitates normal fetal development. Additionally, HTR8-conditioned DCs express low CCR7 even be stimulated by LPS, suggesting that human decidual DCs closest to extravillous trophoblasts may have difficulty homing to the uterine lymph nodes. The data was supported by the study in mice showing that DCs were trapped in deciduas and unable to migrate to lymph nodes^34^. Consist with their phenotypic characteristic, HTR8-conditioned DCs produced high amount of immunoregulatory cytokine IL-10, and limited T cell proliferation, activation and cytokine production. These data indicated a possible mechanism by which EVTs control excessive T activation at the maternal-fetal interface.

Treg cells have been shown to be critical in inducing and maintaining maternal-fetal tolerance. A decreased numbers of Treg is associated with repeated spontaneous abortion in human^35^. Several cell types at maternal-fetal interface are involved in inducing Treg generation, such as decidual stromal cells^36^, NK and macrophages^37^. Recent data indicate that EVTs can directly mediate Treg expansion via secretion of TGF-β, IL-10 and TRAIL^25^. We here showed that DCs differentiated in the present of EVT cell line induce higher Foxp3 expression in naïve CD4^+^T cells compared to control DCs, which in line with previous report showing that DCs cocultured with trophoblast cell line Swan-71 are able to increase the percentage of CD4^+^CD25^+^Foxp3^+^T cells in *ex vivo* DC-T cell interaction^14^. These findings suggest that EVTs may enhance Foxp3 expression on CD4^+^T cells by acting, not only directly on CD4^+^T cells, but also indirectly on dendritic cells progenitors to generate tolerogenic DCs. Of interest, there may be a feedback regulatory loop between decidual tolerogenic DCs and Tregs. The tolerogenic DCs at the maternal-fetal interface express high levels of IDO^38^, and the interaction between CTLA-4 on Tregs and B7 on DCs is involved in mediating IDO expression^39^. This Treg-DC interaction further strengthens the immune tolerance to embryos^38^. In conclusion, the above data reinforce the notion that fetal-derived trophoblast cells are pivotal inducer of maternal immune adaptation.

The underlying mechanisms by which EVT modulate DC differentiation is largely unknown. Our results suggest that the acquisition of a tolerogenic phenotype on DCs induced by EVT is mostly mediated by M-CSF and MCP-1. M-CSF plays a key role in induction of monocytes differentiation. It has been demonstrated by considerable study that M-CSF signaling suppresses the differentiation of monocytes into DCs and redirect the monocytic differentiation to OC/macrophage-lineage cells^21^,^40^,^41^. In the context of pregnancy, M-CSF has been shown to be in high level in the mouse and human uterus during gestation^42^,^43^, which is produced by several cell types such as endometrial stromal cells, uterine NK cells, and trophoblasts^44^,^45^. M-CSF deficiency was related to pregnancy failure caused by Listeria monocytogenes infection^46^. Recent studies showed that M-CSF promote the induction of homeostatic macrophages which shared the phenotype with decidual macrophages^47^. We found significant higher M-CSF in HTR8-DC coculture system, which may counteract the effect of GM-CSF and IL-4, thus skewing the classical DC differentiation toward a tolerogenic profile. We can not rule out the possibility that such cells have local macrophage-like function. The observation that MCP-1 play a role in modulating differentiation of DC is more interesting. MCP-1 is the first identified C-C motif chemokine, involving in recruitment of several leukocytes, such as monocytes/macrophages, T cells, basophils, mast cells and natural killer (NK) cells. It had been reported that MCP-1 protein was expressed by decidual epithelial cells and the first-trimester DSC^48^, and shaped the decidual immune response into Th2 bias^49^. Our very recent study showed the remarkable upregulation of MCP-1 during HTR8 and CD14^+^ monocytes interaction and the promotion of MDSC-like cells by MCP-1^24^. The study focused on the effect of MCP-1 on DC differentiation and function is largely limited. One study reported that MCP-1 by binding of its receptor CCR2 triggered DC maturation, showing increased co-stimulatory molecules expression and enhanced IL-12 production^50^. However, based on our neutralization experiment, high concentration of MCP-1 in HTR8-DC coculture supernatant altered DC differentiation and suppressed IL-12 production by mature DCs. Therefore, MCP-1 may play a distinct role in different stage of DC development. Together, using saturating concentrations of anti-MCP-1 or anti-M-CSF Abs, the two cytokines produced in high concentration in coculture supernatant, we observed large restoration of the phenotypic modifications of DCs differentiation in the presence of EVTs, as well as their ability to produce IL-12p70 upon stimulation by LPS. The fact that M-CSF or MCP-1 antibody even both of them did not fully prevent the tolerogenic effect induced by EVT on the phenotype and function of DCs suggests that factors other than M-CSF and MCP-1 might also be involved.

*In vitro* differentiation of monocyte-derived DC in the presence of HTR8 can replicate some but not all features of *in situ* decidual DC. Because of the low EVT numbers and the lack of proliferative capacity, primary EVTs are difficult to be collected from the tissue and expanded *in vitro*^51^,^52^. A limitation of our study is that all the experiments were performed using HTR8 instead of primary human EVT cells. A very recent report provide a feasible and effective method to isolate primary EVT cells^26^. Further study is planning to carry out to confirm the present observations in this study using primary human EVT cells.

Results presented here provide experimental evidence that human EVT cells, the physiological unit of the placenta, promote the acquisition of a tolerogenic phenotype by DCs, and this effect is mainly mediated by M-CSF and MCP-1. Our study provides significant insights into the regulatory mechanisms behind the interaction of EVTs with the maternal niche at the early stages of gestation.

## Materials and Methods

### Reagents and antibodies

Endotoxin-free reagents and plastic materials were used in all experiments. RPMI-1640 and phosphate-buffered saline (PBS) and fetal bovine serum (FBS)were purchased from HyClone Laboratories (Logan, UT, USA). Recombinant human interleukin-4 (IL-4), recombinant human granulocyte–macrophage colony-stimulating factor (GM-CSF), MCP-1-neutralizing antibody, M-CSF-neutralizing antibody and their isotype-matched control antibodies were purchased from R&D Systems. (Minneapolis, MN, USA). Lipopolysaccharide (LPS) from *Escherichia coli* was purchased from Sigma-Aldrich (St Louis, MO, USA). Antibodies specific for CD14, CD80, CD86, human leucocyte antigen DR (HLA-DR), CD1a, DC-SIGN, CCR7 and their isotype-control antibodies were purchased from BD-Pharmingen(San Diego, CA, USA). The sources of other reagents is indicated in the text.

### Blood sample

The study protocol was approved by Ethics Committee of Qilu Hospital. Blood samples were obtained from healthy non-pregnant donors (n = 20) after providing consent for sample collection and subsequent analysis. The methods were performed in accordance with the approved guidelines.

### EVT Cell Line

HTR-8/SVneo, an immortalized human first-trimester EVT cell line, is derived by transfection of first trimester human trophoblasts with gene encoding simian virus 40 large T antigen described by Graham *et al*.^15^. It was a kind gift from Dr. Charles Graham (Queens University, Kingston, ON, Canada). Cells were maintained in RPMI 1640 supplemented with 10% heat-inactivated fetal bovine serum. All cells were routinely used within 10–50 passages.

### Generation of human DCs

Human peripheral blood mononuclear cells (PBMC) were obtained from blood by Ficoll Histopaque-1077(Sigma-Aldrich, St. Louis, MO, USA) density gradient centrifugation. CD14^+^ cells were isolated using CD14 MicroBeads (MiltenyiBiotech, Bergisch Gladbach, Germany). The process of isolation was performed according to the manufacturer’s instructions. The purity of CD14^+^ cell population was checked by flow cytometry assay and was routinely above 95%. To obtain immature DCs, CD14^+^ monocytes were cultured in RPMI 1640 medium supplemented with 10% FBS, 50 ng/ml GM-CSF and 20 ng/ml IL-4 (R&D Systems) for 6 days. To induce maturation (mDCs), immature DCs were treated with 100 ng/ml LPS (Sigma-Aldrich) for 48 h.

### Co-cultures system

Human extravillous trophoblast cells (HTR-8/SVneo) were initially cultured in 6-well flat-bottom plates in RPMI 1640 medium supplemented with 10% FBS. When the adherent HTR8 reached to 50% confluence, CD14^+^ monocytes were seeded on the top of the HTR8 layer for 3 day in the presence of GM-CSF and IL-4. A titration of HTR8 to monocytes ratio of 1:50, 1:10, 1:5 and1:1 was carried out respectively. On day 3, DCs in the middle of differentiation were transferred to a new prepared HTR8 cell layer, and fresh medium with cytokines was added. At day 5, imDCs were recovered by gently spinning cells in suspension and then washing them. Then imDCs were used for phenotype analysis, or further induced to maturation in the absence of HTR8 cells with 100 ng/ml LPS for an additional 48h. Where indicated, Transwell cambers with a 0.4 μm pore size membrane (Corning) were used to separate monocytes and HTR8 cells. HTR8 cells were cultured in the lower compartment, whereas CD14^+^ monocytes were seeded on the upper compartment.

### Phenotype analysis by FACS

The levels of surface molecules expression on DCs was measured by FITC-, PE-, PE-Cy5-labeled monoclonal antibodies: CD14, CD1a, CD80, CD86, CD83, CCR7 and HLA-DR (BD Pharmingen). Isotype controls were performed in parallel. The samples were acquired on a FACSCalibur flow cytometer, and the results were analyzed by FACS express Version 4.0 software (De Novo Software.CA).

### Detection of cytokines and chemokines

Cell-free supernatants were collected from cultures of imDCs, or HTR8-imDCs at day 5 of differentiation process. The supernatants used for the detection of cytokines were frozen at −80 °C until analysis. Cytokines and chemokines in the supernatants were quantified using the Bio-Plex Protein Array system(Bio-Rad Laboratories, Hercules, CA)as described previously^53^, such as IL-6, IL-10, IL-12p70, IFN-γ, MCP-1, G-CSF and tumour necrosis factor-α (TNF-α). In indicated experiments, M-CSF levels were quantified in cell-free supernatants by ELISA using ELISA kits from R&D Systems and the quantification of the cytokines was performed according to the manufacturer’s instruction.

### CFSE-dilution assays

CD4^+^T lymphocytes were isolated from PBMC by immunomagnetic selection using a naive CD4^+^T cell isolation kit (Miltenyi Biotec) and labeled with CFSE (Invitrogen) in accordance with the manufacturer’s instructions. Briefly, alloreactive T lymphocytes (1 × 10^6^/ml) were stained with 5 μM CFSE. After 10 min, cells were washed twice in PBS with 10% FCS. CFSE-labeled T cells were cultured in the presence of DCs harvested from DC/HTR8 cocultures or those that had been cultured alone during differentiation at a ratio of 5:1. T cells cultured alone were used as negative control, and T cells stimulated by anti-CD3 and anti-CD28 were used as positive control. Flow cytometry was performed on day 5 to assess the proliferating cells.

### T cell activation experiments

Human CD4^+^ naive T cells were isolated from PBMCs by immunomagnetic selection using a naive CD4^+^T cell isolation kit (Miltenyi Biotec). DCs differentiated with or with out HTR8 were extensively washed and incubated with naive allogeneic CD4^+^ cells at a ratio of 1:5. On day 6, the cells were recovered and stained for anti-CD4 and anti-CD69. For intracellular IFN-γ staining, T cells were resuspended in fresh medium and treated with PMA(50 ng/ml)/ionomycin(1 μg/ml)(Sigma-Aldrich) for 6 h and brefeldin A(1 μΜ/ml)(Sigma-Aldrich) for the last 4 h of cultures. The cells were permeabilized and the percentage of CD4^+^/IFN-γ^+^T cells was measured by flow cytometry.

### Induction of regulatory T cells

Human regulatory T cells were induced by DCs as described previously^54^. Human CD4^+^ naive T cells were isolated from PBMCs by immunomagnetic selection using a naive CD4^+^T cell isolation kit (Miltenyi Biotec). Purity of naive CD4^+^T cells was up to 90–95% as confirmed by flow cytometry analysis. Human naive CD4^+^CD45RA^+^T cells (1 × 10^6^) were cultured with allogeneic DCs, or HTR8-conditioned DCs (1 × 10^5^) for 5 days. Then, Foxp3^+^CD4^+^CD25^+^T cells were analyzed by flow cytometry.

### Neutralization of MCP-1 and M-CSF

Neutralizing antibody anti-MCP-1, anti-M-CSF or both were added at day 0 and 3 at the concentration of 20 and 10 μg/ml, respectively (R&D Systems).

### Statistical analysis

All data are shown as mean values ± SEM. One-way ANOVA and the Tukey–Kramer honestly significant difference test was performed to compare multiple groups. In some experiments, two-tailed Student’s *t* test was performed for unpaired samples. All statistical analyses were conducted using GraphPad Prism Version 5.0 (GraphPad Software, CA, USA). A P value of 0.05 was considered statistically significant.

## Additional Information

**How to cite this article**: Zhao, L. *et al*. Human monocytes undergo functional re-programming during differentiation to dendritic cell mediated by human extravillous trophoblasts. *Sci. Rep.* **6**, 20409; doi: 10.1038/srep20409 (2016).
